# Fusion of Audio and Vibration Signals for Bearing Fault Diagnosis Based on a Quadratic Convolution Neural Network

**DOI:** 10.3390/s23229155

**Published:** 2023-11-13

**Authors:** Jin Yan, Jian-bin Liao, Jin-yi Gao, Wei-wei Zhang, Chao-ming Huang, Hong-liang Yu

**Affiliations:** 1 School of Marine Engineering, Jimei University, Xiamen 361021, China; jasonyan@jmu.edu.cn (J.Y.); 18950185198@189.cn (J.-b.L.); hchaoming@jmu.edu.cn (C.-m.H.); 2 Fujian Engineering Research Center of Marine Engine Detecting and Remanufacturing, Xiamen 361021, China; 3 Provincial Key Laboratory of Naval Architecture and Ocean Engineering, Xiamen 361021, China; 4 Information Science and Technology College, Dalian Maritime University, Dalian 116026, China; gao_188@dlmu.edu.cn (J.-y.G.); zhangww@dlmu.edu.cn (W.-w.Z.)

**Keywords:** bearing fault diagnosis, quadratic network, multi-modal signal fusion, audio signal, vibration signal

## Abstract

In this paper, a quadratic convolution neural network (QCNN) using both audio and vibration signals is utilized for bearing fault diagnosis. Specifically, to make use of multi-modal information for bearing fault diagnosis, the audio and vibration signals are first fused together using a 1 × 1 convolution. Then, a quadratic convolution neural network is applied for the fusion feature extraction. Finally, a decision module is designed for fault classification. The proposed method utilizes the complementary information of audio and vibration signals, and is insensitive to noise. The experimental results show that the accuracy of the proposed method can achieve high accuracies for both single and multiple bearing fault diagnosis in the noisy situations. Moreover, the combination of two-modal data helps improve the performance under all conditions.

## 1. Introduction

Bearings, as integral components in various machinery and industrial systems, play a critical role in enhancing operational performance and efficiency. They facilitate reduced friction and smoother operation between moving parts. However, these bearings are prone to wear or damage. If faults are not promptly diagnosed or addressed, it would lead to significant ramifications [1,2].

The presence of faults in rolling bearings can lead to several adverse effects [3,4]. First, faulty bearings can cause a decline in machinery performance and efficiency, resulting in reduced productivity, increased energy consumption, and higher maintenance costs. Second, bearing faults often cause excessive vibration and noise, which not only disrupt the smooth operation of machinery but also cause discomfort for operators and nearby personnel. Thirdly, the vibration and noise generated by faulty bearings can contribute to structural damage in surrounding equipment and infrastructures. Therefore, if bearing faults remain undiagnosed and untreated, they can adversely impact the machine’s performance and the overall system’s functionality.

The implications of bearing faults can be severe and wide-ranging, encompassing diminished equipment performance, increased energy consumption, unexpected downtime, and even catastrophic failure, leading to significant economic losses [5]. Moreover, in sectors where equipment reliability is paramount, such as aerospace, power generation, and transportation, bearing faults can pose serious safety risks [6].

Considering the potential hazards and economic consequences associated with bearing faults, the importance of vibration-based fault diagnosis becomes evident. Vibration analysis is a widely employed technique for monitoring the health condition of rolling bearings due to its sensitivity to changes in the bearing’s dynamic behavior [7]. By analyzing the vibration signals generated by a machine, valuable insights regarding its internal condition, including the presence of bearing faults, can be obtained [8]. This is particularly relevant for bearings, as faults within them often yield characteristic vibration patterns that can indicate specific types of defects [9].

The utilization of audio in bearing fault diagnosis has emerged as a promising approach in recent years. While traditional methods heavily rely on vibration data, audio signals provide supplementary information that can enhance diagnostic accuracy [10]. Audio signals can capture high-frequency components associated with bearing defects [11]. For instance, the presence of a crack or dent in the bearing raceway may produce a distinct acoustic pattern that can be detected and interpreted. This makes audio data particularly valuable for identifying localized faults [12]. Furthermore, audio signals are easy to acquire by microphones with neither complex sensor setups nor direct contact with machinery; therefor, audio-based diagnosis is a potentially less invasive and cost-effective option [13]. Despite these advantages, the utilization of audio in bearing fault diagnosis is still challenging, since audio signals are often susceptible to various environmental noise and interference. Thus, sophisticated signal processing and machine-learning techniques are necessary to extract useful diagnostic information [14].

Existing solutions for bearing fault diagnosis commonly rely on either vibration or audio data analysis. Techniques such as Fourier transform, wavelet transform, and envelope analysis are frequently employed to extract distinctive features from these data, which are subsequently utilized for identifying bearing faults [15,16].

However, these traditional techniques typically focus on a single type of data, either vibration or audio. While these approaches are partially effective, they cannot fully exploit information derived from multi-modal data [17]. For example, certain types of bearing faults may generate characteristic vibration patterns but negligible changes in audio, or vice versa [18]. Consequently, by solely focusing on one type of data, these techniques may overlook crucial fault indicators.

Traditional methods often struggle with the nonlinear and non-stationary nature of time-series fault data, making it challenging to extract distinct fault features [19]. As a result, they may face difficulties when confronted with complex and weak fault signatures, especially in early fault detection where such indicators are subtle and easily overlooked [20].

Advanced machine-learning techniques, particularly deep learning, have shown significant advances in addressing the challenges associated with bearing fault diagnosis. Deep-learning models, such as convolutional neural networks (CNNs) and recurrent neural networks (RNNs), can automatically learn complex patterns and features directly from raw vibration data. This eliminates the need for manual feature engineering and enables more accurate and efficient fault diagnosis [21,22].

In recent years, the fusion of multi-sensor data for fault diagnosis has blossomed into a thriving area of research. A cutting-edge method for diagnosing rolling bearing faults has been proposed [23]. This approach harnesses the power of an advanced multiscale convolutional neural network (CNN), enhanced through the utilization of multi-source data fusion. The innovation in their method lies in the introduction of multiscale convolution kernels, which significantly bolster the model’s resilience against noise. Moreover, they employ global average pooling to preserve the essential details of the feature space. Meanwhile, a novel method has been spearheaded for diagnosing gearbox faults that hinges on multi-sensor deep spatiotemporal feature representation [24]. Spatial data are extracted through parallel CNN, while the power of gated recurrent units (GRU) is harnessed for temporal information retrieval. This approach successfully fuses multi-sensor spatiotemporal data for comprehensive diagnosis. Supplementing these advancements, an intelligent strategy for diagnosing rolling bearing faults has been presented in [25]. It fuses multiple signals with a Morlet transform function-residual network (MTF-ResNet). Multi-source signals are efficiently combined using an image fusion framework, temporal correlations are captured via the MTF, and intricate fault features are extracted by deploying an optimized ResNet model.

To summarize, great advances have been achieved in fault diagnosis, but there are still several limitations. For instance, information provided by single modality is limited, and commonly utilized deep-learning networks need a huge amount of parameters to achieve complex mapping functions [26,27]. In the evolving landscape of bearing fault diagnosis, quadratic convolutional neural network (QCNN) has emerged as a transformative tool. Unlike traditional CNN, QCNN with its inherent quadratic neurons offers enhanced parameter efficiency and superior representation of intricate data patterns, making it effective in detecting subtle changes in signals. It is insensitive to noisy environments and the introduction of the ‘Qttention’ mechanism further bolsters its interpretability and accuracy. Therefore, QCNN is theoretically a robust and reliable diagnostic tool. As industry conditions strive for efficient and interpretable diagnostic solutions, QCNN stands out as a promising contender, ensuring machinery safety and reliability [28,29].

In essence, by integrating the advantages of multi-modal data fusion and advanced machine learning, the proposed method holds significant promise in offering a more accurate, reliable, and efficient solution for bearing fault diagnosis.

This paper is organized as follows. The importance and challenges of diagnosing bearing faults, as well as related works, are provided in Section 1. The preliminaries, including the quadratic network and attention mechanism, are presented in Section 2. Then, the proposed method is presented in detail in Section 3, including the audio-vibration signal fusion, fusion feature extraction model, and decision model. After that, the experimental results and discussions are elaborated in Section 4. Finally, the conclusions are drawn in Section 5.

## 2. Preliminaries

### 2.1. Quadratic Network

#### 2.1.1. Quadratic Neurons

In deep learning, conventional neural networks consist of layers of neurons, which use an inner product of the inputs with a nonlinear activation function before outputting the results. In order to enhance the representation ability of conventional neurons, the quadratic neuron was introduced. The quadratic neuron replaces the inner product with a quadratic function of the input vector, which upgrades the first-order neuron to the second-order neuron, empowers individual neurons, and facilitates the optimization of neural networks [30].

The computation process contained in a quadratic neuron is shown as below:

(1) $$\sigma (f(x))=\sigma \left(\left(\sum_{i=1}^{n}w_{i}^{r}x_{i}+b^{r}\right)\left(\sum_{i=1}^{n}w_{i}^{g}x_{i}+b^{g}\right)+\sum_{i=1}^{n}w_{i}^{b}x_{i}^{2}+c\right)=\sigma \left((x^{T}w^{r}+b^{r})(x^{T}w^{g}+b^{g})+{\left(x⊙x\right)}^{T}w^{b}+c\right)$$

where the input vector is transformed into two inner products and one norm term for summation before the nonlinear activation, σ(⋅) is a nonlinear activation function, ⊙ denotes the Hadamard product, $w^{r}$, $w^{g}$, $w^{b}\in {\mathbb{R}}^{n}$ are weight vectors, respectively, and $b^{r}$, $b^{g}$, c∈ℝ are biases, respectively. The superscripts r, g, and b are just marks for convenience without special implications.

#### 2.1.2. Superior Representation

Compared to the conventional neuron, the improvement with regard to the representation ability of the quadratic neuron is intrinsic, since it is the involved nonlinear computation which contributes to the improvement rather than the increased parameters.

In addition, quadratic neurons have been proven to be powerful in fitting more complicated functions owing to the employment of a quadratic aggregation function. Compared to conventional neurons which only obtain nonlinear mapping from the nonlinear activation function, quadratic neurons obtain an additional nonlinear mapping. As a result, a single quadratic neuron can realize XOR logic which cannot be achieved by the conventional neurons. On the other hand, a quadratic neuron is not equal to the combination or summation of three conventional neurons. The combination of conventional neurons can only be a piecewise linear function, whereas a quadratic neuron is a piecewise polynomial function. Therefore, quadratic neurons exhibit a better performance in terms of expressiveness due to the fact that a polynomial spline is better at approximating complicated functions than a linear spline.

### 2.2. Attention Mechanism

The attention mechanism was proposed to address problems that are encountered when tackling with long sequences in deep learning. Conventional RNN and CNN do not work well in capturing important features in long sequences. The attention mechanism can capture important features efficiently by dynamically calculating the contribution to the output of each position, therefore improving the performance of the model. The following definition of the attention mechanism and the symbols used are consistent with those in [31].

We denote the input matrix for our entire model as $X\in {\mathbb{R}}^{d_{x}\times n_{x}}$, where $d_{x}$ represents the size of the input vectors and $n_{x}$ represents the amount of input vectors. The input matrix X is then, by passing through the feature model, transformed into the $n_{f}$ feature vectors $f_{1},\ldots ,f_{n_{f}}\in {\mathbb{R}}^{d_{f}}$, where $d_{f}$ represents the size of the feature vectors.

Considering the fact that the attention model is to emphasize specific parts of the input features, a query vector $q\in {\mathbb{R}}^{d_{q}}$, with $d_{q}$ indicating the size of the query vector, is introduced to determine which part of the feature vectors is of interest.

The feature vectors $F=[f_{1},\ldots ,f_{n_{f}}]\in {\mathbb{R}}^{d_{f}\times n_{f}}$ and the query vector $q\in {\mathbb{R}}^{d_{q}}$ are jointly used as input for the attention model, the goal of which is to selectively focus on the most relevant parts of the input data while ignoring the irrelevant parts.

The attention model consists of a single or a collection of general attention modules, which aim to generate a weighted average of the input. To achieve this, we first obtain two matrices, the keys matrix $K=[k_{1},\ldots ,k_{n_{f}}]\in {\mathbb{R}}^{d_{k}\times n_{f}}$ and the value matrix $V=[v_{1},\ldots ,v_{n_{f}}]\in {\mathbb{R}}^{d_{v}\times n_{f}}$ where $d_{k}$ and $d_{v}$, respectively, indicate the dimensions of the key and value vectors. These two matrices are obtained through linear transformations of F using weight matrices $W_{K}\in {\mathbb{R}}^{d_{\kappa }\times d_{f}}$ and $W_{V}\in {\mathbb{R}}^{d_{v}\times d_{f}}$ for K and V, correspondingly, as

(2) $$\begin{array}{c} K=W_{K}\times F\\ V=W_{V}\times F \end{array}$$

After that, the weights $e=[e_{1},\ldots ,e_{n_{f}}]\in {\mathbb{R}}^{n_{f}}$, which are inherently involved in computing a weighted average, are obtained by combining the query q with the keys matrix K using a score function denoted as score(). The calculation is performed as

(3) $$e_{l}=\mathrm{score}(q,k_{l})$$

where $e_{l}$ represents how important the information contained in the key vector $k_{l}$ is according to the query.

Next, a normalization technique is employed to ensure that the weights are mapped to the range [0, 1]. Given that the objective of an attention module is to yield a weighted average of the input, the resulting weights are further adjusted using an alignment function called align(), which is shown as

(4) $$a_{l}=\mathrm{align}(e_{l};e)$$

where $a_{l}\in \mathbb{R}$ is the attention weight corresponding to the l-th value vector.

The outputs of the attention model, denoted as $c\in {\mathbb{R}}^{d_{v}}$, are referred as the context vectors because they capture the relationships between the current feature vector $f_{i}$ and other counterparts in F. The context vectors are obtained by calculating the weighted average of the columns of the value matrix V with each column being assigned a weight $a_{l}$. The calculation is shown as

(5) $$c=\sum_{l=1}^{n_{f}}a_{l}\times v_{l}$$

The context vector c is then utilized by the output model to make the final prediction $\hat{y}$. A common approach to generate the output using c is to apply a softmax function on it as

(6) $$\hat{y}=\mathrm{softmax}\left(W_{c}\times c+b_{c}\right)$$

where $d_{y}$ is the number of output choices or classes, and $W_{c}\in {\mathbb{R}}^{d_{y}\times d_{v}}$ and $b_{c}\in {\mathbb{R}}^{d_{y}}$ are trainable weights.

## 3. Proposed Method

To utilize complementary information of audio and vibration signals, and to extract compact features for bearing fault diagnosis, we propose a dual-channel audio-vibration signal fusion method based on a quadratic convolution neural network. This method comprises three stages: an audio-vibration signal fusion model, fusion feature extraction model, and decision model. The input signals are the raw audio and vibration signals acquired under identical conditions, while the output is the fault diagnosis result. The proposed method enables end-to-end bearing fault diagnosis and is to be presented in detail in this section.

### 3.1. Audio-Vibration Signal Fusion

To effectively utilize information of audio and vibration signals in the time domain, it is necessary to fuse the signals from these two channels together. Therefore, we adopted a 1 × 1 convolution to accomplish this task. The 1 × 1 convolution can fuse the features from the two channels while maintaining the size of the feature. Moreover, it can learn the non-linear relationship between the audio and vibration signals.

Mathematically, given the input audio signal $S\in {\mathbb{R}}^{L\times 1\times 1}$ and vibration signal $V\in {\mathbb{R}}^{L\times 1\times 1}$, they are viewed as two separate channels of the overall observation; then, we obtain the input vectors $I\in R^{L\times 1\times 2}$ whose two channels are composed of S and I, respectively. After that, the input vectors are passed through the 1×1 convolution layer and ReLU activation function. Therefore, the output of the signal fusion model can be obtained as

(7) $$I^{\prime }=\sigma_{R}(W\cdot I+b)$$

where $\sigma_{R}(\cdot )$ is the ReLU activation function, and W is the weight vector of the 1 × 1 convolution layer.

### 3.2. Fusion Feature Extraction Model

The fused feature $I^{\prime }$ is obtained through the audio-vibration signal fusion model. To achieve the fast and accurate fault diagnosis of bearings, it is important to obtain compact and comprehensive features. Therefore, a quadratic convolutional neural network (QCNN) [29] is used as the feature extraction module. The QCNN implicitly contains an attention mechanism. QCNN is also a simple and effective network which performs stably and competitively under various noise levels. Compared to other models, QCNN features a smaller model size, lower computational complexity, and shorter inference time. Quadratic neural units in QCNN inherently involve nonlinear transformations, thereby resulting in additional nonlinear mappings.

**Figure 1:**
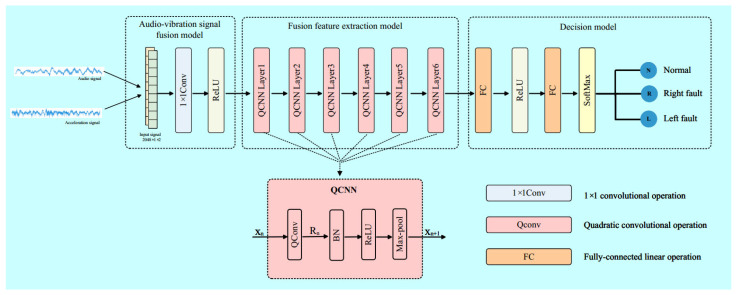
Overview of the proposed method.

As shown in Figure 1, the audio-vibration fusion feature extraction module consists of six layers, each containing a quadratic convolutional neural network.

Let $x_{n}$ be the input vector for the n-th QCNN, as depicted in Figure 1. Thus, we can obtain

(8) $$R_{n}=Q(x_{n})$$

where $Q(x_{n})$ is the output of QCNN with input $x_{n}$.

According to Equation (1), we can obtain

(9) $$R_{n}=\left((x_{n}^{T}w_{n}^{r}+b_{n}^{r})(x_{n}^{T}w_{n}^{g}+b_{n}^{g})+{\left(x_{n}⊙x_{n}\right)}^{T}w_{n}^{b}+c_{n}\right)$$

where $w_{n}^{r}$, $w_{n}^{g}$, and $w_{n}^{b}\in {\mathbb{R}}^{n}$ are weight vectors, respectively, and $b_{n}^{r}$, $b_{n}^{g}$, and $c_{n}\in \mathbb{R}$ are biases, respectively.

As demonstrated in [29], Equation (10) can be simplified to be

(10) $$R_{n}=x_{n}^{T}(x_{n}⊙w_{n}^{b}+w_{n}^{g}x_{n}^{T}w_{n}^{r}+w_{n}^{g}b_{n}^{r}+w_{n}^{r}b_{n}^{g})$$

The bias term $w_{n}^{g}b_{n}^{r}+w_{n}^{r}b_{n}^{g}$ in the above equation can be excluded since it is irrelevant to $x_{n}$. The term $x_{n}⊙w_{n}^{b}+w_{n}^{g}x_{n}^{T}w_{n}^{r}$ in the QCNN intrinsically exhibits the attention mechanism [29]. That is to say, the attention in QCNN is not realized by extra computations, but it originates from the quadratic neurons.

Then, the output of the n-th QCNN is

(11) $$X_{n+1}=\phi (\sigma_{R}(\beta (R_{n})))$$

where ϕ(⋅) and β(⋅) are max pooling and batch normalization operators, respectively.

### 3.3. Decision Model

The feature extraction module generates a high-dimensional vector comprising features of fusion signals. Therefore, dimension reduction is required for accurate bearing fault diagnosis. The decision module conducts the dimension reduction through two fully connected layers before obtaining the specific bearing fault diagnosis results as

(12) $$e=f(W_{r}x+b_{r})$$

(13) $$y=\mathrm{Softmax}\{[W_{f}\sigma_{R}(e)+b_{f}]\}$$

where $W_{r}$ and $W_{f}$ are the weight vectors of the fully connected layers, respectively; $b_{r}$ and $b_{f}$ are the biases of the fully connected layers, respectively.

## 4. Experiment

In this section, we will elaborate the experimental results and discussions in detail.

### 4.1. Dataset Description

In this study, we focus on the 204EM cylindrical roller bearing, which is a single row bearing capable of handling high radial loads and operating at high speeds. It has dimensions of 20 mm (inner diameter), 47 mm (outer diameter), and 14 mm (width), with 11 rollers, each having a diameter of 7.5 mm. The bearing’s pitch diameter is 34 mm, and its contact angle is 0°, which is typical for cylindrical roller bearings where line contact occurs between the rollers and the raceways. One of its defining characteristics is the presence of two integral flanges on the outer ring, while the inner ring lacks flanges, allowing axial displacement in both directions. Additionally, the bearing features a separable design, facilitating mounting and the interchangeability of components. With its high radial load carrying capacity, low friction, long service life, and ability to accommodate axial displacement, the 204EM serves as an excellent model for studying bearing performance and failure modes under different operational conditions.

**Figure 2:**
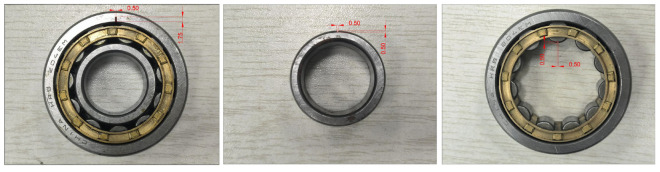
Three types of bearing faults.

To simulate bearing failure, we utilized the electrical discharge machining (EDM) technique to introduce faults into the outer race of the 204EM cylindrical roller bearing. Initially, controlled EDM was applied to the outer race, involving rapid electrical discharges between the bearing and an electrode to cause localized melting and vaporization of the material. By adjusting parameters such as discharge current, pulse duration, and electrode shape and size, the desired fault was created. Subsequently, the bearing underwent a meticulous inspection to verify that the artificially induced fault matched the desired characteristics. An intentional defect, with dimensions of 1.75 mm in depth and 0.5 mm in width, was engineered on the inner side of the bearing’s outer ring using EDM. A defect was intentionally engineered on the inner side of the bearing’s outer ring using EDM, with dimensions of 1.75 × 0.5 mm (depth × diameter). To further analyze the vibration responses of different bearing component faults, defects were additionally introduced in the inner race and rolling element of the 204EM cylindrical roller bearing using the EDM technique. For the inner race defect, controlled electrical discharges were applied to create an intentional fault with dimensions of 0.5 × 0.5 mm (depth × width) on the outer side of the bearing’s inner ring. For the rolling element defect, a similar EDM process was utilized to produce an artificial defect sized 0.5 × 0.5 mm on the surface of a roller. The bearings containing these extra induced faults were carefully examined to validate the desired flaw dimensions and locations. The three types of bearing faults are displayed in Figure 2.

Following the successful introduction of the fault, the bearing was installed and operated under various conditions to simulate different operational scenarios. Vibration and noise measurements closely monitored the bearing’s performance, and the collected data were subsequently analyzed to assess the effect of the induced fault on its operation.

In this study, we used a single-axis accelerometer (The ‘Model 333B30 accelerometer was sourced from PCB Piezotronics, Inc., located in Depew, New York, USA) as part of our experimental setup to capture vibration data. Additionally, an ICP sound pressure sensor (The microphone used in our experiments, a ‘1/2” model’, is manufactured by GRAS Sound & Vibration A/S, based in Holte, Denmark) was utilized to collect noise data, providing supplementary information regarding the operational state of the bearing. These sensors were connected to an LMS SCADAS data acquisition system from SIEMENS, which converted the analog signals into digital data for subsequent analysis. To facilitate equipment control, data collection, and analysis, a DELL precision m3800 notebook (DELL, Round Rock, TX, USA) workstation served as the central hub. This integrated approach allowed us to comprehensively examine the bearing’s performance and failure modes under various conditions. Table 1 provides an overview of the measurement equipment used in the study.

**Figure 3:**
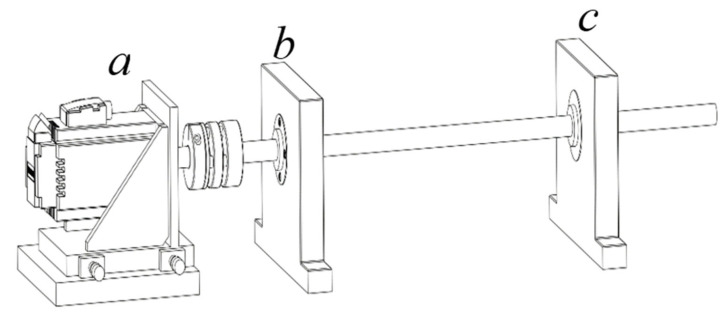
Experimental setup schematic.

As depicted in Figure 3, the experimental setup comprises several key components. Part a shows the stepper motor, which is controlled by the control cabinet to provide rotational motion to the shaft. The shaft, central to the setup, is supported by two bearings at distinct positions: the left bearing, depicted in part b, and the right bearing, shown in part c. This configuration allows for the analysis of the bearings’ performance under different load and speed conditions, offering a comprehensive evaluation of their functionality.

**Figure 4:**
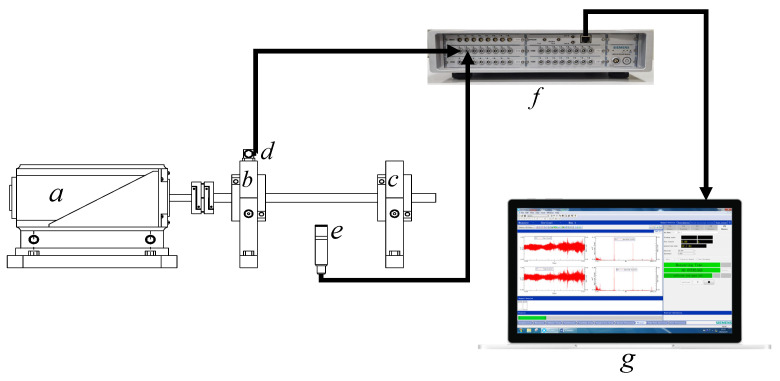
Schematic diagram of the test setup.

As shown in Figure 4, part a shows the stepper motor, a crucial component of the experimental setup. Part b represents the left bearing, where the accelerometer sensor (part d) is mounted. The sound pressure sensor, denoted as part e, is positioned between the left bearing (part b) and the right bearing (part c). Signals collected from these sensors are transmitted to the LMS SCADAS Data Acquisition System, illustrated in part f. Data processing and result display are then carried out on a DELL Notebook Workstation, shown as part g.

**Figure 5:**
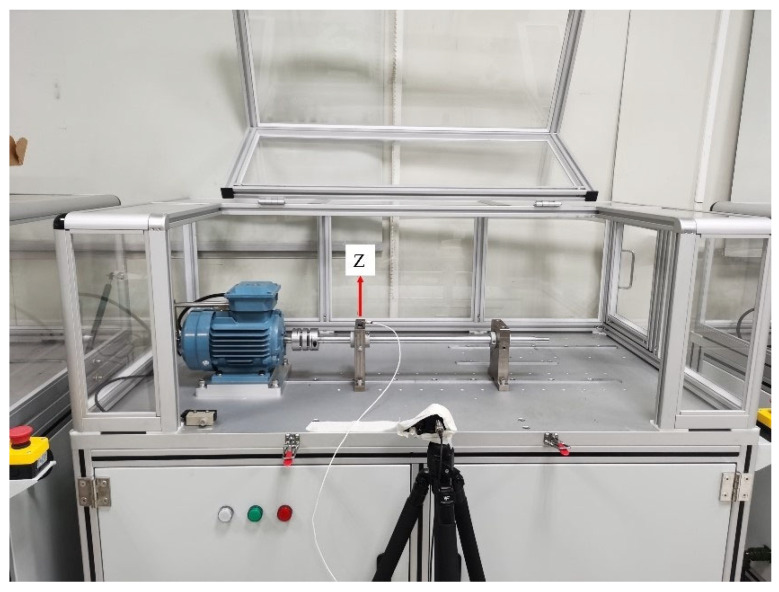
Bearing experiment setup for RBF_16384 dataset.

For the bearing fault dataset (called RBF_16384 from now on), a bearing was used to support a shaft for collecting vibration and audio signals under working speeds of 1000 RPM, 1600 RPM, and 2000 RPM, respectively. The locations of the vibration and audio sensors are illustrated in Figure 5. The direction of the uniaxial accelerometer from PCB, used for vibration detection, is indicated in this figure. The ‘Z’ axis represents the direction perpendicular to the surface of the bearing seat. The audio signal was captured using a Gras microphone (The microphone used in our experiments, a ‘1/2” model’, is manufactured by GRAS Sound & Vibration A/S, based in Holte, Denmark). Seven types of vibration and audio signals were recorded, including the normal state, left outer race fault, right outer race fault, left inner race fault, right inner race fault, left rolling element fault, right rolling element fault, and inner race fault, at a sampling rate of 16,384 Hz. The duration for each condition was 15 min.

For these datasets, we used the same faulty bearings and equipment placement for data collection, with only different working speeds. The purpose of varying the working speeds was to verify the model’s ability under different operational conditions and to assess the impact of rotational speed on detecting bearing faults. The eight states are represented by the labels N, LO, RO, LI, RI, LE, and RE for normal, left outer race fault, right outer race fault, left inner race fault, right inner race fault, left rolling element fault, and right rolling element fault, respectively, as shown in Table 2.

### 4.2. Data Preprocessing and Parameter Setting

We sequentially cut the audio and vibration signals into short sequences of a length of 2048. Since they are recorded simultaneously at the same condition, the short sequences of audio and vibration signals with the same index are combined together and assigned with one-hot labels.

Cross-entropy was chosen as the loss function, and the stochastic gradient descent [32] was used to optimize our network with a batch size of 64. The network includes six QCNN layers, with the first QCNN layer containing a convolutional kernel of size 64 × 1 and the remaining five layers containing convolutional kernels of size 3 × 1. For the RBF_16384 dataset, the learning rate is 0.025. For the RBF_102k dataset, we chose a learning rate of 0.005. Additionally, in the QCNN network, we need to set α to adjust the learning rate of the quadratic term. α was set to be 0.03. Meanwhile, Gaussian white noise was mixed with the original signal to simulate the performance of the model in a noisy environment. The signal-to-noise ratio (SNR) was set as 5 dB. The SNR is defined as

(14) $$SNR=10\log_{10}\frac{P_{S}}{P_{N}}$$

where $P_{S}$ is the average power of signal and $P_{N}$ is the average power of noise.

All experiments were conducted in Windows 11 with an Intel i7 12650H CPU at 2.30 GHz and one NVIDIA RTX 4060 8 GB GPU. The code was written in Python 3.8 under the framework of PyTorch.

### 4.3. Results and Discussions

There are three experiments to evaluate the performance of the proposed method. In more detail, they were designed for the following purposes: (1) to determine whether the proposed method can discriminate the right-side, left-side, and normal states at different working speeds; (2) to determine whether it can discriminate different types of bearing fault in a noisy condition; (3) to determine whether QCNN is better than CNN, which is a simpler approach with only linear convolution in a noisy condition. They will be elaborated in detail in the section.

The data are divided into segments of 2048 points, and we obtained 7200 samples for each working speed of each mode (a total of 151,200 samples for three categories). The raw samples are input into the network for processing; therefore, the bandwidth is 8192 Hz (equal to half of the sampling rate). For the rotation speed of 1000 RPM, each rotation takes 0.06 s. Thus, in one frame with 2048 points (0.125 s long), we can capture about 2.08 full rotations. For the rotation speed of 1600 RPM, each rotation takes 0.0375 s. Thus, there are approximately 3.33 full rotations for each frame. For the rotation speed of 2000 RPM, it takes 0.03 s per rotation, resulting in around 4.17 full rotations for each frame. As can be seen, for the given RPMs, our data length is adequate to capture multiple complete rotational cycles. This ensures obtaining the full information during the bearing rotations.

#### 4.3.1. Results and Discussions of Experiment 1

**Figure 6:**
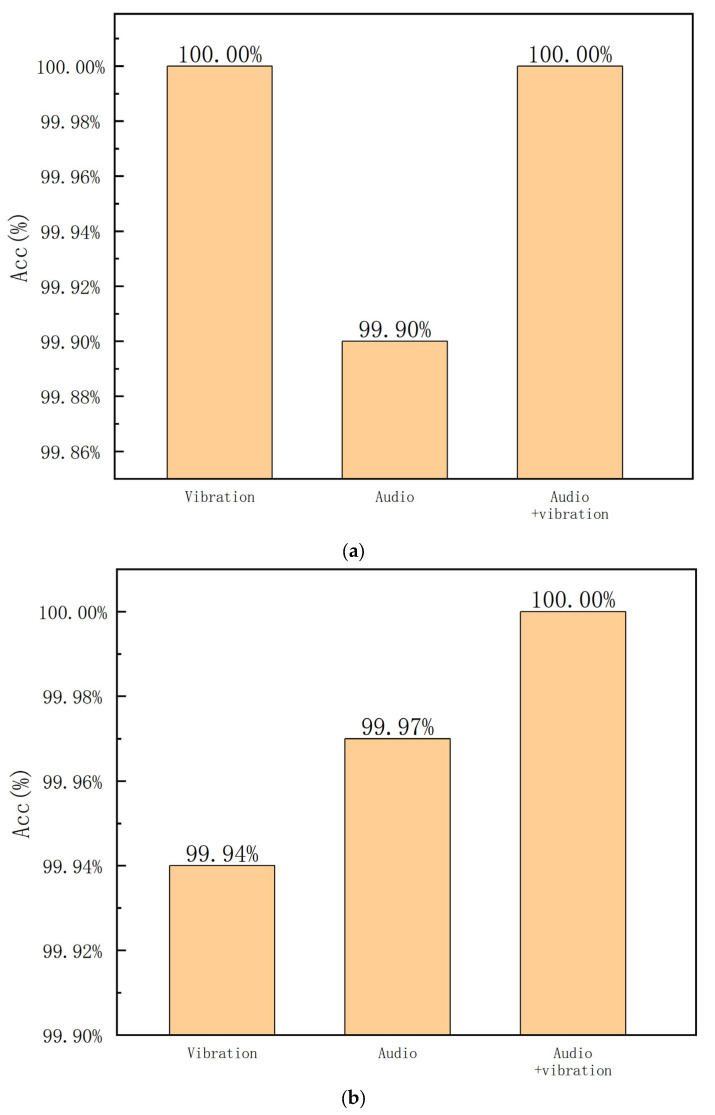

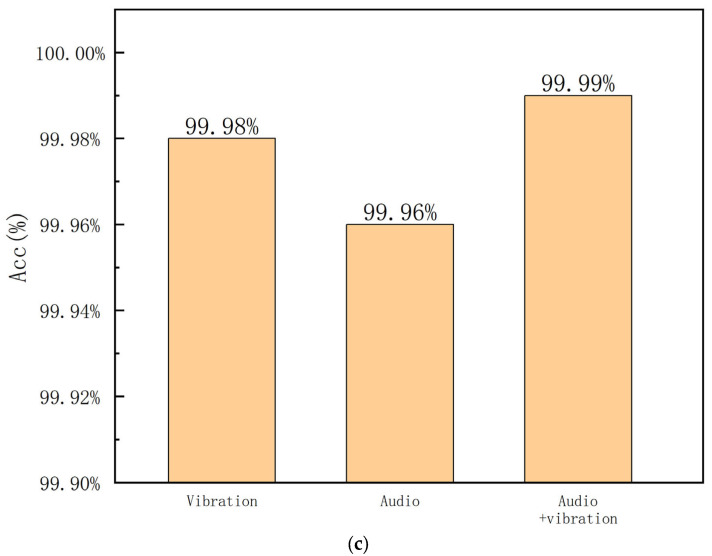
Accuracies for the outer race fault under different working speeds; (**a**) 1000 RPM; (**b**) 1600 RPM; (**c**) 2000 RPM.

First, we examined the performance of the proposed method for a single type of bearing, i.e., the bearing with an outer race fault. To compare the performance of different input signals, we input three kinds of signals, i.e., vibration, audio, and audio-vibration. The experimental results are provided in Figure 6. It can be seen that the quadratic neuron in feature extraction is very powerful. Additionally, the performance of the audio signal as input is slightly inferior to vibration. And the combination of vibration and audio signals helps to improve the fault diagnosis performance. The accuracies of the proposed method with these three inputs are all high, indicating that the proposed method is effective.

**Figure 7:**
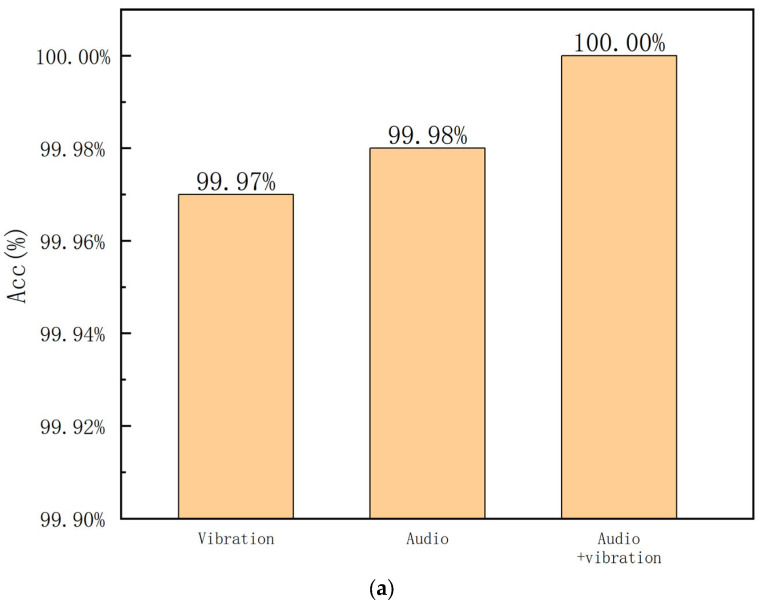

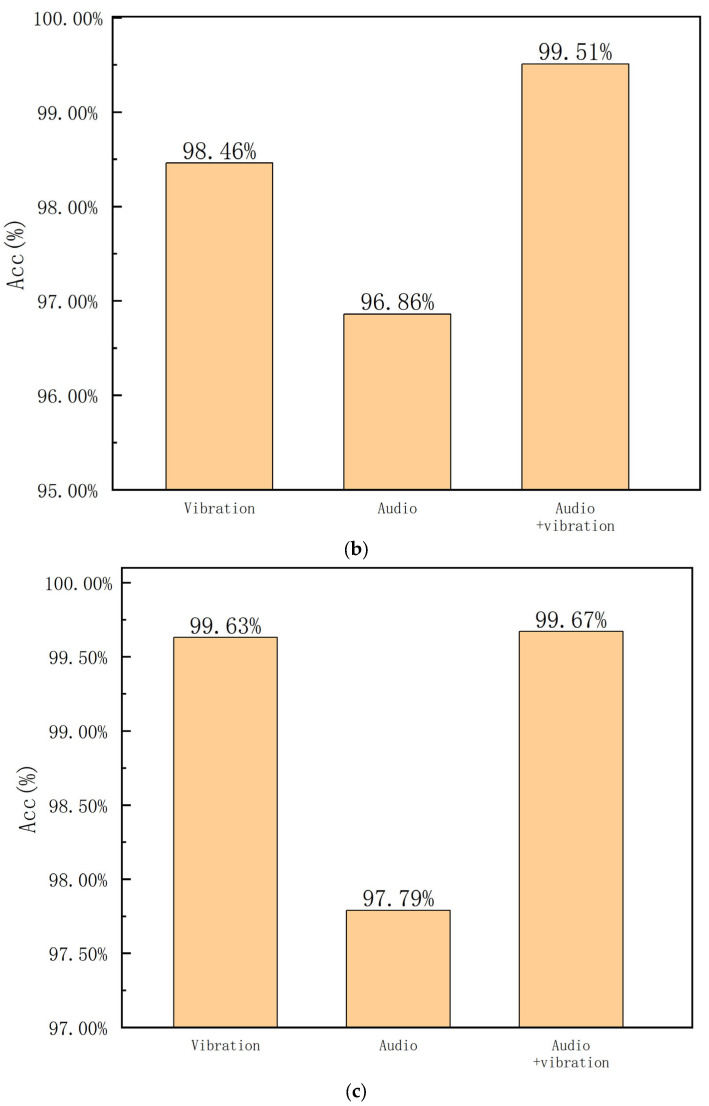
Accuracies for the outer race fault with a 5 dB SNR under different working speeds; (**a**) 1000 RPM; (**b**) 1600 RPM; (**c**) 2000 RPM.

To verify the performance of the proposed method in the noisy conditions, the collected signals are mixed with white noise with an SNR equal to 5 dB. The input signals are the same as the first experiment. The accuracies are provided in Figure 7. It can be seen that the situation with the vibration signal as input is sensitive to noise, and the combination of both vibration and audio signals is more robust to noise. The evaluation results also confirm that the fusion of two modality signals, i.e., vibration and audio, is effective.

**Figure 8:**
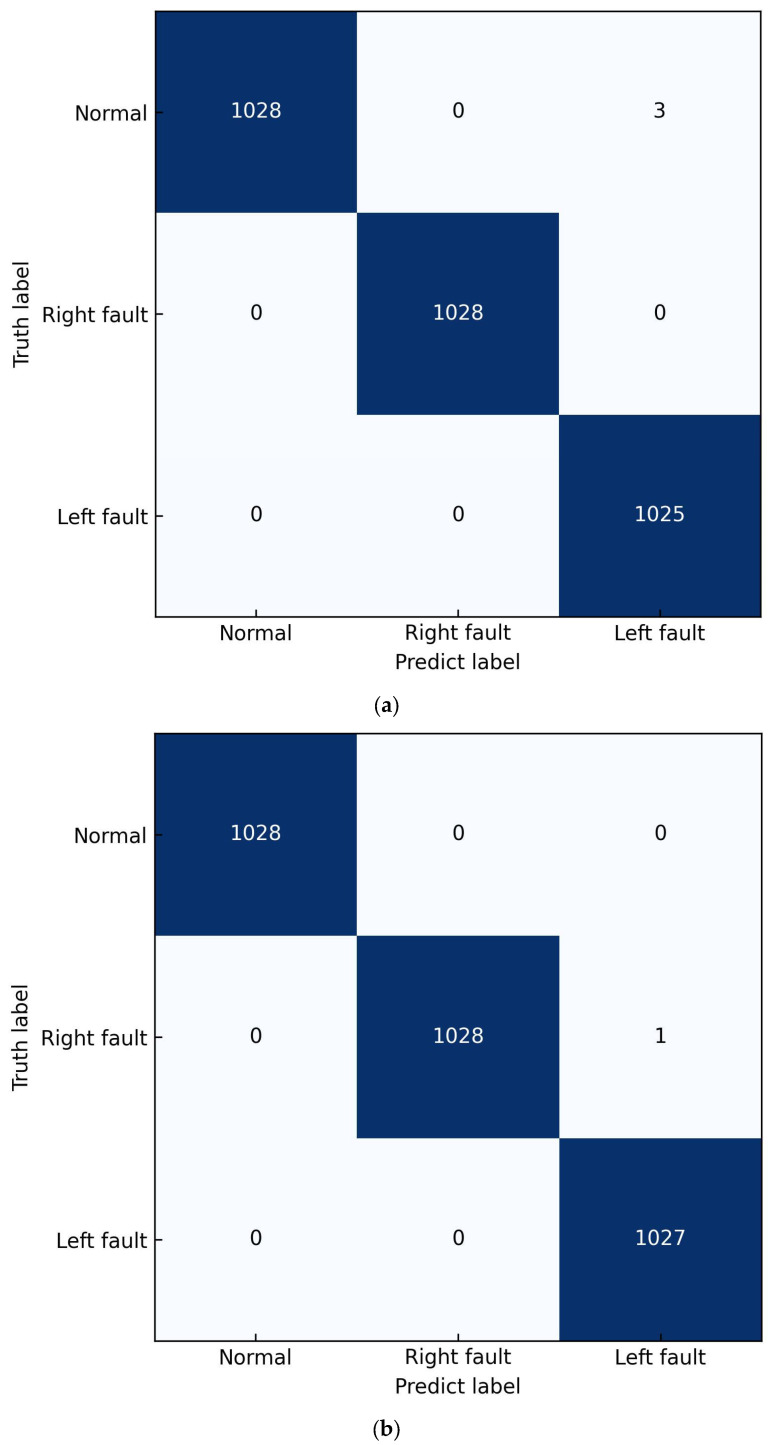
Confusion matrices on different input signals; (**a**) Confusion matrix with audio signal as input at a 5 dB SNR; (**b**) Confusion matrix with vibration signal as input at a 5 dB SNR.

Moreover, in the experiment with a 5 dB SNR, the model with a single audio signal as input misclassifies three left-side fault samples into normal mode, and the model with signal vibration signal as input misclassifies a left-side fault as a right-side fault, as shown in Figure 8a,b, while the model with dual-channel signals as input could classify all samples correctly. Therefore, even though a single vibration or audio signal as input could achieve a high accuracy, it is not perfect and there are still incorrect judgements. However, the two-modality signal as input can achieve a higher accuracy by utilizing the complementary information of both of the two modalities.

**Figure 9:**
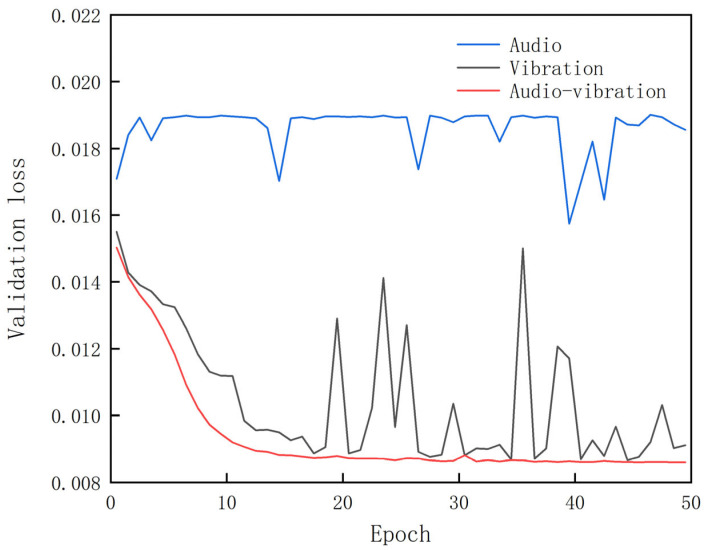
Loss curves on validation set.

The loss curves on the validation set are given in Figure 9. We can find that the validation set loss of the model trained on audio signals remains very high. For the model trained on vibration signals, even though the loss steadily decreases with the increase in epoch, it occasionally fluctuates, indicating that the loss is not always stable. This phenomenon did not occur when using a dual-channel signal input, and when we added residual connections to the single-channel vibration signal model, the fluctuations were a bit lower. This suggests that the robustness of the model has been improved by using dual-channel inputs and adding residual connections. It can also be seen that the loss with both audio and vibration signals as input decays the fastest, indicating the superiority of fusion of both signals.

**Figure 10:**
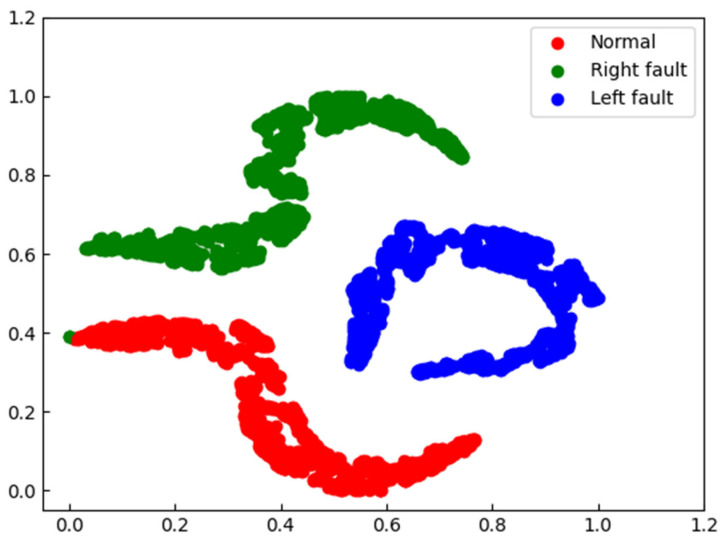
t-SNE results of features produced by the last convolutional layers with a vibration signal model.

**Figure 11:**
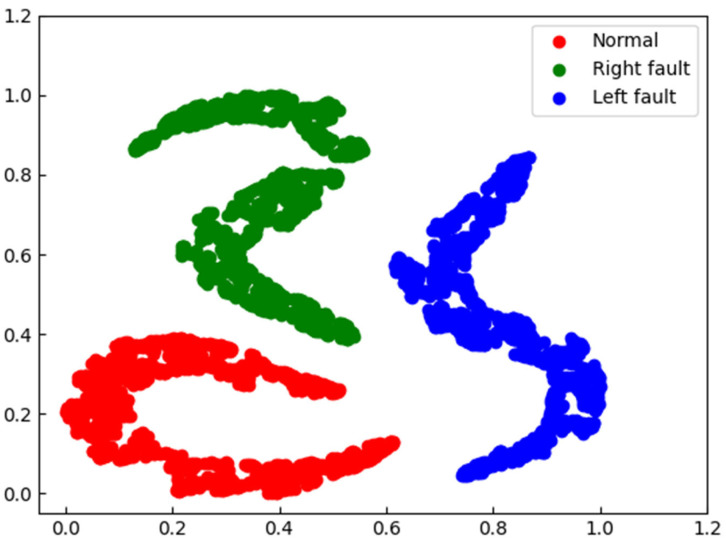
t-SNE results of features produced by the last convolutional layers with an audio-vibration signal model.

In order to further compare the features learned from single-channel and audio-vibration inputs, we used t-distributed stochastic neighbor embedding (t-SNE) [33] to visualize the output features of the last convolutional layer for single-channel vibration signal input and audio-vibration input, as shown in Figure 10 and Figure 11, where different colors represent different fault categories of the bearing. We can observe that when the input is the single-channel vibration signal, the red cluster representing the normal bearing is contaminated by the green cluster representing the faulty bearing on the right side, while in the dual-channel input, the red and green clusters are well separated, indicating that the dual-channel input has a better identification ability for bearing faults.

#### 4.3.2. Results and Discussions of Experiment 2

To test whether the proposed model can deal with more sophisticated situations, we extended the training and test set in the first experiment such that they contain four bearings, with a left outer race fault, right outer race fault, left inner race fault, right inner race fault, left rolling element fault, right rolling element fault, and normal state, respectively. To further improve the difficulty of the fault diagnosis, white noises was added into the signals with an SNR equal to 0 dB. All the samples were mixed together to form the training set. We have also captured the same types of faults at a different time to constitute the test set.

The evaluation results are provided in Table 3. As illustrated in this table, the accuracies that take the audio signal as an input are higher than those with vibration signals as the input. This observation indicates that the audio signal is more robust than the vibration signal in complex situations. Moreover, the accuracies with both audio and vibration signals as an input are higher than those with either modal signal as the input. Finally, the proposed method can achieve at least 98.81% in the complicated situation.

In summary, the evaluation results demonstrate that the proposed method can deal with sophisticated situations, and the combination of both audio and vibration signals can improve the accuracies. 

#### 4.3.3. Results and Discussions of Experiment 3

To verify if QCNN is better than CNN, we replaced the QCNN with CNN, and conducted the second experiment again: the accuracies are provided in Table 4. It can be observed that most accuracies in Table 4 are lower than the corresponding ones in Table 3. And the accuracies with both modality signals as inputs range from 71.96% to 97.86%. This experiment indicates that QCNN can indeed better model the mapping function from the input signal to fault diagnosis.

## 5. Conclusions

In this paper, a quadratic convolution neural network for an end-to-end diagnosis of bearing faults is utilized. The audio and vibration signals are first fused together by a 1 × 1 convolution and passed through a QCNN to make use of the complementary information of these two modalities. Then, the QCNN extracts the complex features from the input audio and vibration signals. Finally, the decision model deduces the fault diagnosis result. The experimental results demonstrate that the sample-level bearing fault diagnosis accuracies of the proposed method for single-bearing fault diagnosis achieve 99.99% for different working speeds, and they can exceed 99.51% in the noisy situations with a signal-to-noise ratio of 5 dB. The accuracies for the multiple-bearing fault diagnosis exceed 98.81% when taking both audio and vibration signals as input. The experimental results demonstrate that QCNN outperforms CNN in modeling the mapping function from input signals to fault diagnosis, achieving a higher diagnostic accuracy with multimodal signal inputs. With the combination of more than two samples, the proposed method can achieve 100% for both situations.

## Figures and Tables

**Table 1:** Experimental conditions.

Equipment Name	Model	Manufacturer	Sensitivity
Single-axis Accelerometer	333B30	PCB	10.14 mV/(m/s^2^)
ICP Sound Pressure Sensor	1/2″	GRAS	50 mV/Pa
Computer	Notebook Workstation	DELL	-
Data Acquisition System	LMS SCADAS	SIEMENS	-

**Table 2:** Seven types of labels in our datasets.

Label	Fault Mode
N	Normal
RO	Right outer race fault
LO	Left outer race fault
LI	Left inner race fault
RI	Right inner race fault
LE	Left rolling element fault
RE	Right rolling element fault

**Table 3:** Accuracies of the proposed method in discriminating different types of bearing fault. White noises is added into the signals with an SNR equal to 0 dB.

Bearing Type	Input Signal	Accuracy of the Proposed Method
Outer race	Audio	98.32%
	Vibration	98.80%
	Audio + Vibration	99.03%
Inner race	Audio	83.49%
	Vibration	78.77%
	Audio + Vibration	99.93%
Rolling element	Audio	85.26%
	Vibration	48.08%
	Audio + Vibration	98.81%
Normal	Audio	98.01%
	Vibration	6.07%
	Audio + Vibration	99.95%

**Table 4:** Accuracies of the proposed method with QCNN replaced by CNN. White noises is added into the signals with an SNR equal to 0 dB.

Bearing Type	Input Signal	Accuracy of the Proposed Method
Outer race	Audio	95.31%
	Vibration	96.33%
	Audio + Vibration	97.86%
Inner race	Audio	98.76%
	Vibration	76.85%
	Audio + Vibration	79.67%
Rolling element	Audio	9.34%
	Vibration	73.57%
	Audio + Vibration	71.96%
Normal	Audio	29.52%
	Vibration	98.93%
	Audio + Vibration	79.67%

## Data Availability

The data presented in this study are available on request from the corresponding author.
